# Perinatal programming - myths, fact, and future of research

**DOI:** 10.1186/s40348-014-0002-2

**Published:** 2014-09-04

**Authors:** Jörg Dötsch

**Affiliations:** Department of Pediatrics, University of Cologne, Kerpener Str. 62, Köln, 50937 Germany

**Keywords:** Perinatal programming, Thrifty phenotype, Mismatch hypothesis, Epigenetics, Metabolic disease, Diabetes mellitus

## Abstract

**Background and Findings:**

Perinatal programming, i.e., the (epigenetic) modification of (genetic) functions throughout lifetime, suffers from the notion of premature theories and difficult and extensive research strategies.

**Conclusions:**

This mini review aims at depicting 9 current developments and discusses possible future research strategies.

## Introduction

When, in 1991, thrifty phenotype hypothesis [1] was formulated, it appeared that an old concept was revived: the ability of an individual to react to environmental changes with an adaptive response, i.e., limits the supply to organs that are utmost importance and delays the development of systems not urgently needed. However, there is a price to pay: The neglected organs become insufficient later, and life and diseases such as diabetes mellitus type 2 become more prevalent in that group (Hales and Barker, [2]).

The initial discovery was followed by an extensive search for diseases more prevalent in persons who were born small for gestational age. Many conditions were found to be associated such as cardiovascular disease, metabolic syndrome, diabetes mellitus, renal disease, cancer, and even psychiatric disorders. The spectrum of intrauterine influences leading to postnatal alterations was increased; the influence of overnutrition in the womb, psychosocial stress, high salt intake, and many more were scrutinized; and a tremendous load of original and review publications was produced [3].

Almost 25 years after the first publications, the mini review will focus on three key issues:

1. What are the current concepts of perinatal programming? Will it be possible to achieve a unifying concept?
2. Do we have enough insight into potential mechanisms of perinatal programming?
3. Where are the pitfalls of current research? Can we develop new strategies?

### Current concepts of perinatal programming (Figure 1)

#### From the thrifty phenotype (Barker-) hypothesis to the mismatch hypothesis

Several criticisms were raised soon after the thrifty phenotype hypothesis was inaugurated: first, the increased risk for morbidity later in life after being born with a high birth weight had been neglected. This was soon corrected, and nowadays, intrauterine overfeeding is regarded as a major risk factor for cardiovascular and metabolic disease [4]. Second, the postnatal environment was found to be of utmost importance leading to the creating of the so-called mismatch hypothesis, indicating that the discrepancy between intrauterine and postnatal nutrition determines the later phenotype [5]. However, the mismatch hypothesis fails to explain why children with intrauterine overnutrition experience an increased later morbidity risk if they receive continuous overnutrition after birth [6].

**Figure 1:**
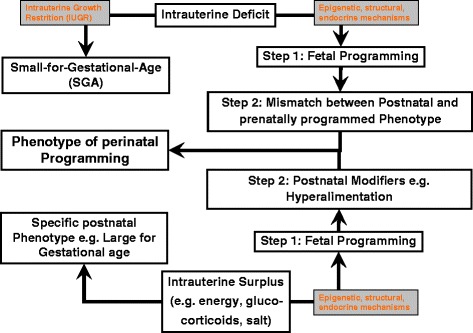
**Concept of fetal and perinatal programming the mismatch hypothesis may only be applied to the intrauterine deficit situation, not to the intrauterine surplus.**

#### Is there a unifying concept?

As a consequence Plagemann suggests an alternative, unifying concept arguing that perinatal programming should not be regarded as a coping strategy to actively compensate developmental conditions but rather a vegetative learning process leading to passive adaptations of the organism [6]. In detail, three key fields interact with each other and form the phenotype of perinatal programming and the developmental origins of health and disease. These are the following: (1) natural and social environment, (2) epigenomic plasticity, and (3) microstructural plasticity. In particular, these adaptations are not necessarily ‘aiming’ at improving an organism situation in a teleologic sense [4].

### Mechanisms of disease

It is now widely recognized that the mechanisms leading to perinatal programming are epigenetic in nature. Epigenetic changes are alterations of genomic function not modifying gene structure as such. Whether they all lead to DNA modifications will be discussed in this section.

#### Altered gene expression

Gene expression can be altered by several mechanisms influencing mRNA transcription. The most important ones are DNA methylation, histone modification, and noncoding RNAs, most of which is known from animal and cell culture studies [7]. In the last 5 years, at least 20 human studies have shown associations between in utero exposition and an altered DNA methylation of certain genes. In most cases, the effect of nutrient supplements such as folic acid was examined; however, several studies have addressed intrauterine deficiency (Tobi et al. [8,9]). Overexposition as in maternal diabetes mellitus has also been shown to inflict changes in gene methylation [10]. Despite these progresses in understanding the potential mechanisms of perinatal programming, the exact effects of changes in gene methylation are not always easy to assess.

#### Other mechanisms?

The earliest mechanistic observations that were made were structural changes in organs that are altered by perinatal programming. One example in that context is the kidney, where already years ago, a reduction in nephron number was demonstrated after intrauterine growth restriction [3]. This was well in line with a study showing that reduced nephron number is associated with hypertension [11].

Another example for structural changes is the alteration of the hippocampal structure and function by perinatal programming in the context of stress and nutrition. As a consequence, memory, endocrine, and metabolic consequences emerge [12]. A classic experiment in that context showed that nerve fibers needed for energy and appetite regulation originating in the arcuate nucleus of the hypothalamus depend on the presence of leptin in a critical time window [13].

It is not entirely understood whether these structural changes are secondary to modifications in the function of developmental genes and how they are inflicted on a mechanistic basis.

Apart from structural alterations, endocrine adaptations are important in a mechanistic sense to explain the consequences of perinatal programming. The hypothalamic-pituitary-adrenal axis is probably the best characterized target. Others are the 11β hydoxysteroid dehydrogenase in the kidney and adipose tissue and the growth hormone insulin-like growth factor axis. The impact of these changes can be seen in an increased stress responsiveness, arterial hypertension, or generalized or local alterations of growth. Again, the link to epigenetic changes is obvious [14].

### Potential research strategies

#### Limitations of actual research

There are several limitations and pitfalls in the research of perinatal programming.

Human studies suffer from the disadvantage that the exact intrauterine exposure to a programming event such as nutrient supply cannot easily be determined. Low or high birth weight is a poor surrogate of the exact intrauterine events. Documentation of intrauterine growth or placental function is better, however still far from an exact mechanistic insight. Therefore, huge cohorts have to be examined to achieve a study power high enough. Some epidemiological studies therefore have populations of several million participants [15]. In addition, most of the outcome parameters (such as diabetes mellitus type 2, coronary heart disease) only occur later in adult life. Not only this increases the study period to an almost impossible time, but also the number of confounders that may become apparent during a life span is immense. As a consequence, many studies use surrogate instead of hard end point parameters, always leading to the question whether the study is really valid.

Laboratory and animal studies apparently overcome those two major disadvantages. It is possible to differentiate various causes of surplus and deficit situations. As an example, protein deficiency (mimicking undernutrition in the developing countries) leads to a different endocrine phenotype than ligation of the uterine arteries, simulating placental insufficiency [16]. In addition, the outcome can be scrutinized more thoroughly than in clinical studies. Also animal experiments are very attractive with regard to the possibility to examine potential mechanisms in detail.

Nonetheless, apart from the well-known difficulties to transfer data to humans, some pitfalls have to be addressed: Frequently, male and female animals show a completely different phenotype. The exact causes of the gender influence are not well understood. Also, since usually not a single gene is responsible, the number of animals needed may be very high and it is even less certain, whether results may be transferred to humans than in diseases where a single gene or a well-defined mechanism is responsible.

#### Future research

Unanimously, therefore, most scientist advocate studies with larger human cohorts, starting early in pregnancy or even before, gain as much information as possible on the exact background and mechanism of the presumed programming event and an integration of bio sampling to address potential mechanisms [17,18]. The disadvantage of the long study duration cannot be easily solved and demands large consortia and a potent and long-lasting financing situation. Possibly, a large number of additional secondary objectives may be integrated facilitating the emergence of a consortium [19].

As to animal studies, the choice of the appropriate species and intervention model is of utmost importance as depicted above [20]. A greater emphasis should be put on the use of transgenetic animals to get nearer to the underlying mechanisms of perinatal programming. Transgenic models could help to evaluate the significance of single genes or pathways in the evolvement of the programmed phenotype.

## Conclusions

Research in the field of perinatal programming suffers from several drawbacks: some potentially premature theories that are presently being further developed and the need for extremely large and costly studies. Nonetheless, diseases having their origin in utero and leading to diseases only very much later in life bear the opportunity to be addressed during a critical time window. Therefore, research strategies should adapt to these needs.
